# Supplementary material for: Cargo Recognition of Nesprin‑2 by the Dynein Adapter Bicaudal D2 for a Nuclear Positioning Pathway That Is Important for Brain Development
Source: Biochemistry. 2026 Mar 2;65(6):717–31. doi: 10.1021/acs.biochem.5c00596 (PMC13001094; doi:10.1021/acs.biochem.5c00596)
Supplement: Supplementary file 1 [file bi5c00596_si_001.pdf]

SUPPORTING INFORMATION

Cargo recognition of Nesprin-2 by the dynein adapter Bicaudal D2 for a nuclear positioning pathway that is important for brain development

Estrella D Rodriguez Castro[$,1], Sivasankar Putta[$,1], M. Yusuf Ali[$,2], Jose M Garcia Martin[1], Xiaoxin Zhao[1,◆], Samantha Sylvain[1], Kathleen M Trybus[*,2] and Sozanne R Solmaz[*,1]

[1] Department of Chemistry, Binghamton University, PO Box 6000, Binghamton, NY 13902, USA.

[2] Department of Molecular Physiology and Biophysics, University of Vermont, Burlington, VT 05405, USA.

[$]First authors, equally contributed. *Corresponding authors.

SRS: E-mail: ssolmaz@binghamton.edu

Phone: +1 607 777 2089; ORCID 0000-0002-1703-3701

KMT: E-mail: Kathleen.Trybus@med.uvm.edu

Phone: +1 802-656-8750; ORCID 0000-0002-5583-850

◆*X.Z. deceased on October 18, 2023*

Table S1. Summary of contact residues from PISA. Experimentally validated contact residues are denoted by an asterisk.

Nesprin-2/BicD2<br>Nesprin-2<br>aa 6123-6421	Nesprin-2/BicD2<br>BicD2<br>aa 715-804	Rab6/BicD2<br>BicD2<br>aa 715-804	Nup358/BicD2<br>BicD2<br>aa 715-804
6124			732
6160	734		
6164			735
6171			736
6174			737
6175	738		
6178			739
6181			740
6182	741		741
6185	742		742
6189			743
6193			744
6195	745		
6241	746		746
6242			747
6245	749		749
6246*			750
6249			751
6250*	753		753
6252			754
6253*			755
6254	756		756
6256	757		757
6257			758
6259			759
6260*	760		760
6261*			761
6312			762
6315			763
6319*			764
6322	765		765
6323			766
6326			767
6329*			768
6333			769
6337			770
	772		772
	774	774	774
	775		775
			776
	777	777	
	778	778	
	779	779	779
	781	781	781
		782	782
	783*	783	783
	784	784	
	785	785	785
	786*	786	786
	787	787	
	788*	788	788
	789*	789	789
	790	790	790
	791		
	792	792	
	793	793	793
	794	794	
	795	795	795
	796		
	797*	797	797
		798	
	800		
	801	801	801
	804	804	

See also Figure 3.

Table S2. Secondary structure estimation from the CD spectra with BeStSel [1]

Nesprin-2 mutant	α-helix (%)	Anti-parallel β-sheet (%)	Parallel β-sheet (%)	Turn (%)	Other (%)
**WT**	51.9	0	0	11.2	36.9
**V6246A**	45.6	1.8	1.9	12.0	38.6
**E6250A**	48.4	8.3	0.1	11.3	31.9
**H6260A**	48.8	0	0	11.1	40.1
**V6246A/L6253A/F6261A**	52.7	1.8	0	9.7	35.8
**R6329A**	52.2	15.6	0	19.4	12.7
**Y6319A**	48.1	2.2	0	9	40.8

Table S3. Nesprin-2 phosphorylation sites from the PhosphoSitePlus v 6.8.1 database

Phosphorylation Site (human)	Phosphorylation Site (mouse)	References
S6314	S6301	[2]
T6348	T6335	[3]
S6349	S6336	[3–5]
T6351		[5]
S6361	S6348	[6]
T6365	T6352	[7]
		[8]
S6384	S6371	[2]
	T6375	[9]
S6389	S6376	[8]
S6392	S6379	[9,10]
	S6400	[9]
	S6403	[9]

Note that the minimal BicD2 binding site was mapped to aa 6123 - 6352 of mouse Nesprin-2, which is followed by the adaptive domain (aa 6353 - 6421).

Table S4. Nesprin-1 phosphorylation sites.

Nesprin-1 phosphorylation sites with known kinase specificity from the PhosphoSitePlus v 6.8.1 database

Phosphorylation Site (human)	Phosphorylation Site (mouse)	Kinase	Reference
T8033		**PLK1**	[11–13]

Nesprin-1 phosphorylation sites from the PhosphoSitePlus v 6.8.1 database

Phosphorylation Site (human)	Phosphorylation Site (mouse)	Source
T8033		[11–13]
S8223	S8227	[14]
S8232		[15]
S8236	S8240	[15]
S8250	S8254	[5]
S8270		[16]
S8274	S8278	[9,16,17]
S8277	S8281	[18]
S8280	S8284	[18]
S8305	S8308	[6]

Note that the BicD2 recruiting SR70 – SR71 are located in aa 7998 -8216 of mouse Nesprin-1, which is followed by the adaptive domain.

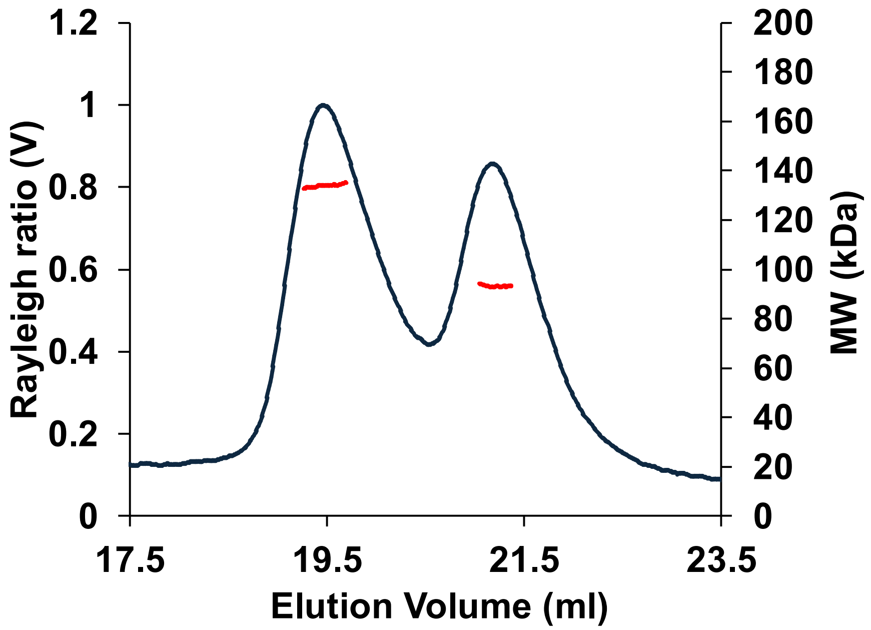

Figure S1. Size exclusion chromatography coupled to multi-angle light scattering (SEC-MALS) data suggest a 2:2 stoichiometry for the minimal Nesprin-2/BicD2 complex. 0.225 mg/ml Nesprin-2 (GST-tagged, aa 6123-6352; for sequence see Figure S13) and 0.04 mg/ml BicD2-CTD (aa 715-804, the his_6-tag was proteolytically cleaved) were mixed and incubated for 30 min prior to SEC-MALS analysis. The Rayleigh ratio (blue) and weight-averaged molar masses (MW in kilodaltons, red) are plotted versus the elution volume (ml) for a representative experiment. Molar masses were determined across the selected peak area and plotted on a secondary axis. The average MW from four experiments is MW=128.9 ± 4.9 kDa for the left peak, which closely matches the calculated mass of a 2:2 complex of Nesprin-2 and BicD2 (130.4 kDa; note that the mass of a BicD2 protomer is 10.9 kDa, and the mass of a Nesprin-2 protomer is 54.3 kDa). For the right peak, MW= 94.1 ± 2.2 kDa. The standard deviation was calculated as error.

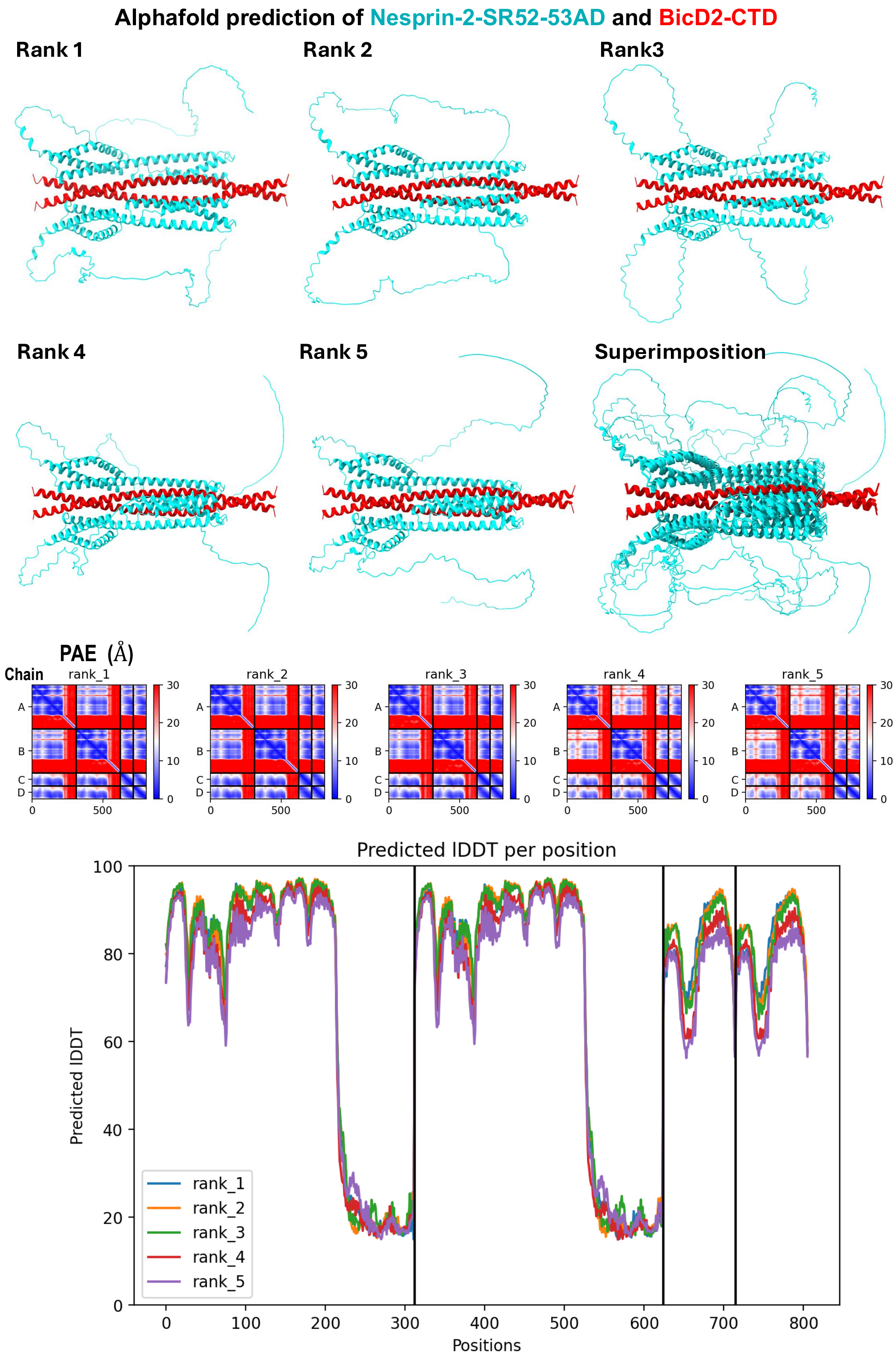

Figure S2. AlphaFold prediction of the minimal Nesprin-2/BicD2 complex (Nesprin-2-SR52-SR53AD and BicD2-CTD). The five highest ranked predicted structures are shown individually and superimposed in cartoon representation, together with the PAE and pLDDT error plots. See also Figure 1.

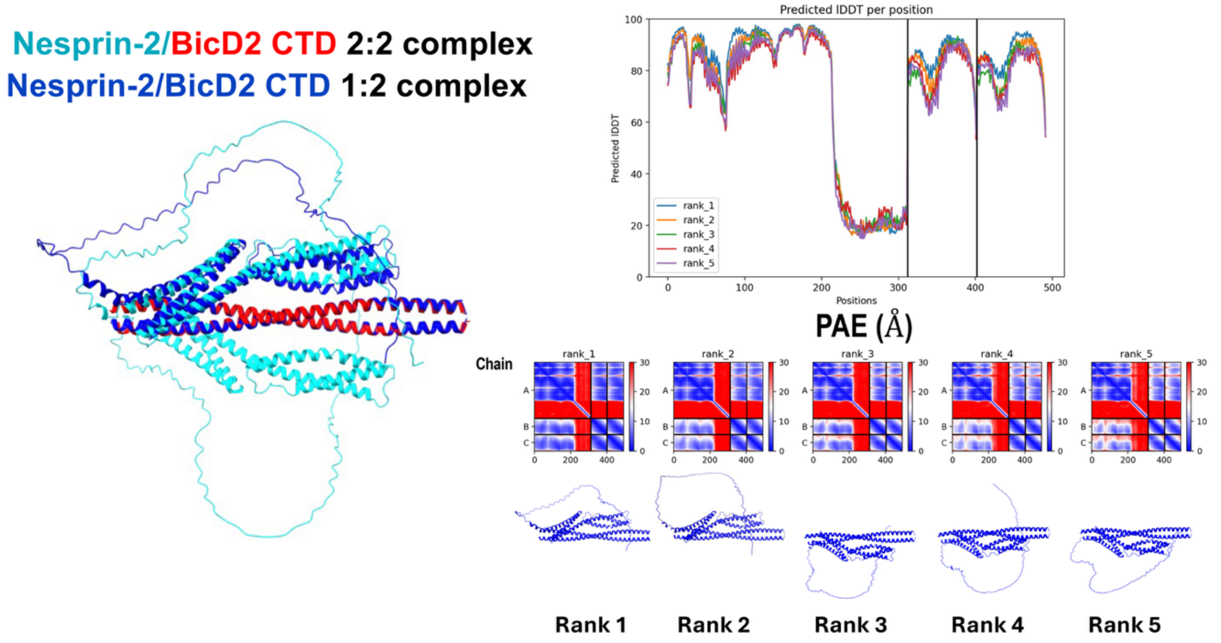

Figure S3. AlphaFold prediction of the minimal Nesprin-2/BicD2 complex with a single Nesprin-2 molecule bound to two BicD2 molecules in blue (Nesprin-2-SR52-SR53AD and BicD2-CTD). The prediction is least-squares superimposed with the 2:2 complex of Nesprin-2 and BicD2 in cyan and red. The RMSD is 1.8 Å, which suggests that the predicted binding mode of a single Nesprin-1 is very similar to the binding mode of two Nesprin-2 molecules (note that the disordered regions have the highest RMSD). The cartoon representations of the five highest-ranking models are shown along with their PAE and pLDDT error plots. See also Figure 1.

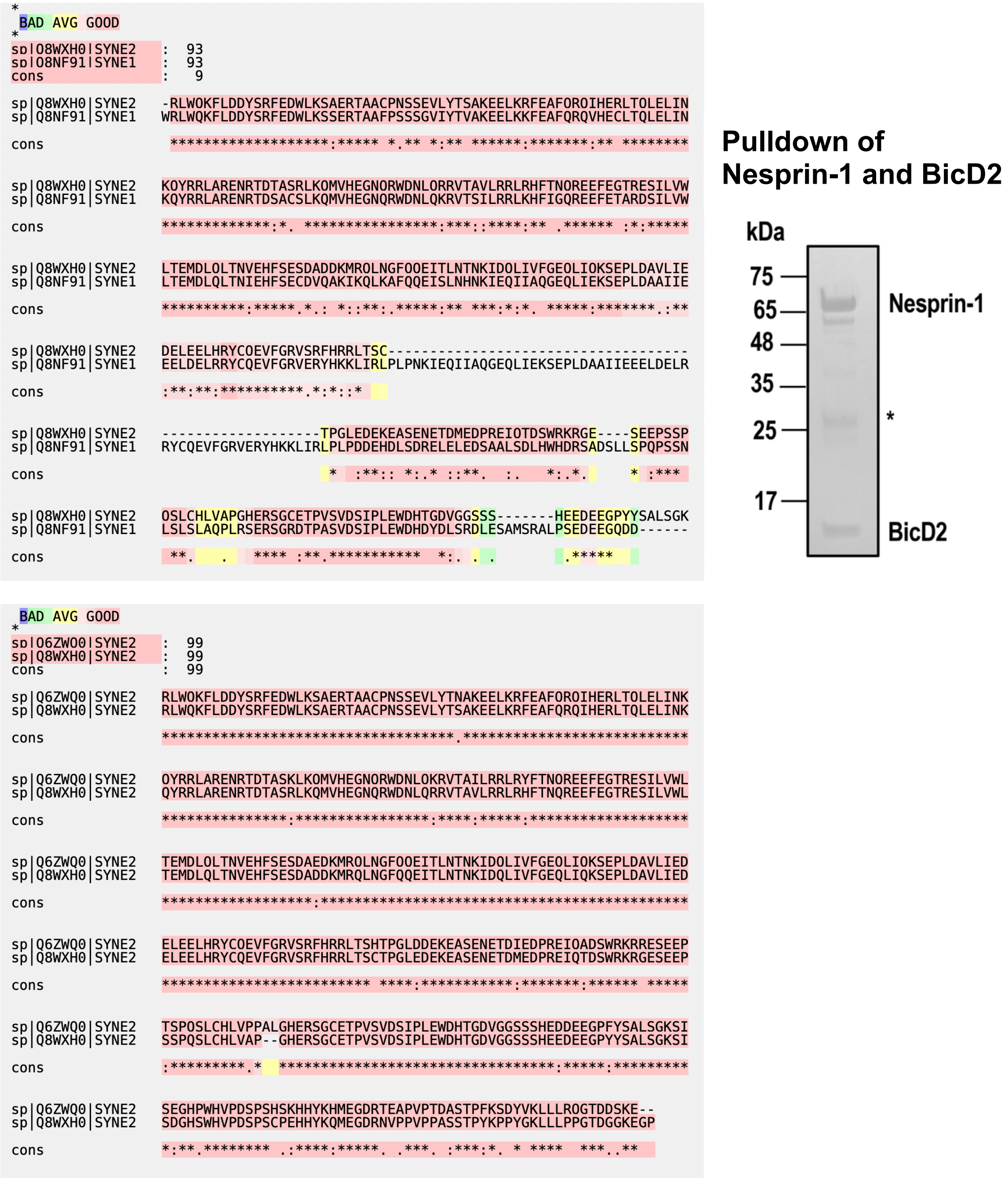

Figure S4. Top: A sequence alignment of human Nesprin-2-SR52-SR53AD and human Nesprin-1-SR70-SR71AD confirms high sequence conservation (prepared with T-COFFEE, version 11.00). SDS PAGE analysis of the elution fraction from a pull-down assay confirms that the GST-tagged Nesprin-1-SR70-SR71AD-GST fragment interacts with the His$_6$-tagged BicD2-CTD. The asterisk denotes GST. Masses of standard proteins are indicated**.** Bottom: **sequence alignment of mouse and human Nesprin-2-SR52-SR53AD**. Uniprot accession numbers for the sequences are shown. The sequence conservation (cons) is illustrated by a color gradient from red (good) to blue (bad), for which the scale is shown at the top, together with the overall sequence conservation. Asterisks denote identical residues, colons denote highly conserved residues, full stops indicate partially conserved regions and blank spaces denote residues that are not conserved. See also Figure 1.

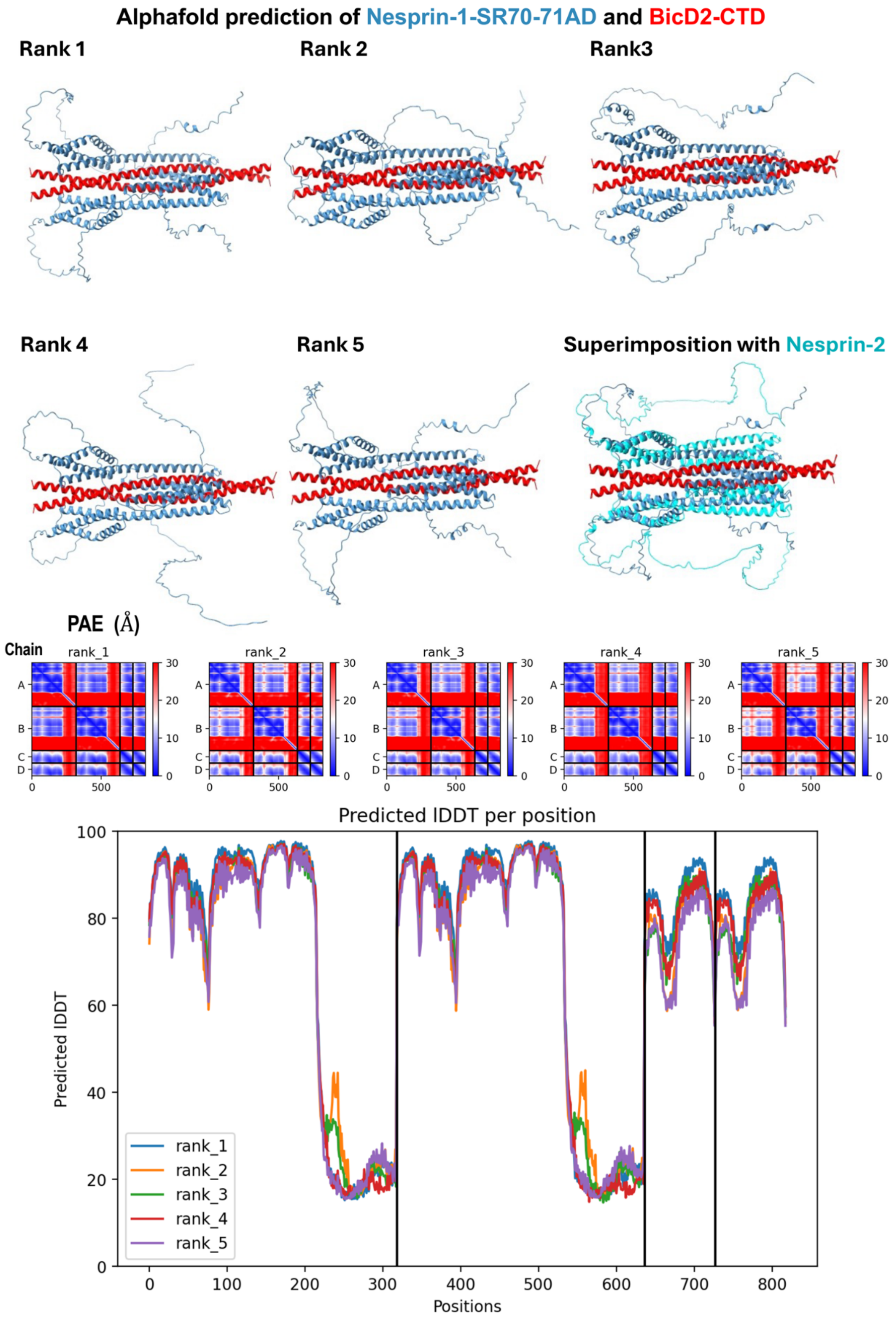

Figure S5. AlphaFold prediction of the Nesprin-1-SR70-SR71AD and BicD2-CTD complex. The five highest ranked predicted structures are shown in cartoon representation, together with the PAE and pLDDT error plots. See also Figure 1.

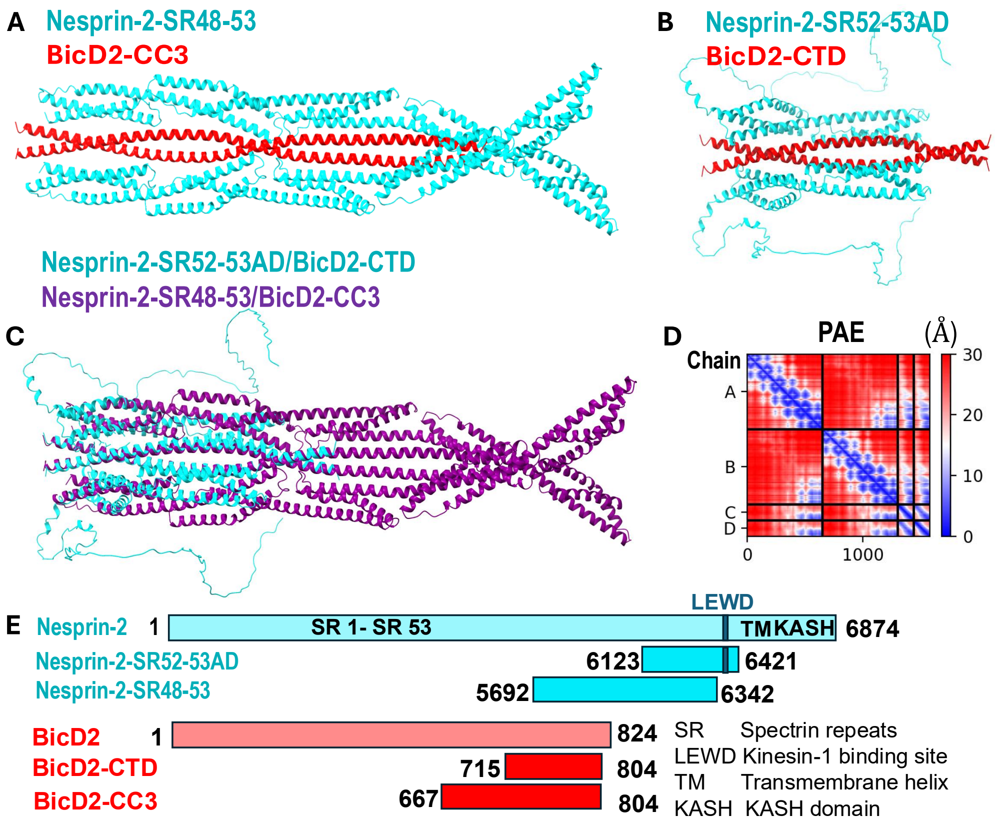

Figure. S6. The predicted structure of a larger Nesprin-2/BicD2 complex including SR 48- SR 58 and the entire coiled coil 3 of BicD2 supports the predicted structure of the minimal complex. (A) Structural model of a larger Nesprin-2/BicD2 complex (Nesprin-2 aa 5692-6342 [19] and BicD2 aa 667-804) from AlphaFold, shown in cartoon representation. (B) Structural model of the minimal complex (Nesprin-2 aa 6123-6421 and BicD2 aa 715-804). (C) Least squares superimposition of A (purple) and B (cyan) (RMSD = 0.99 Å). Note that the predicted structure of the minimal complex closely resembles the predicted structure of the same domains in the context of the larger complex. (D) Predicted aligned error (PAE) plot for the highest-ranking model. The x and y axes show the residue numbers, starting with Nesprin-2 (Chain A, B), followed by BicD2 (Chain C, D). Note that the high PAE suggests that the predicted structure of the additional regions in the larger complex is not reliable. Additional models and error plots are shown in Figure S7. (E) Schematic representation of the Nesprin-2 and BicD2 fragments used for A and B [19–21]. See also Figure 1.

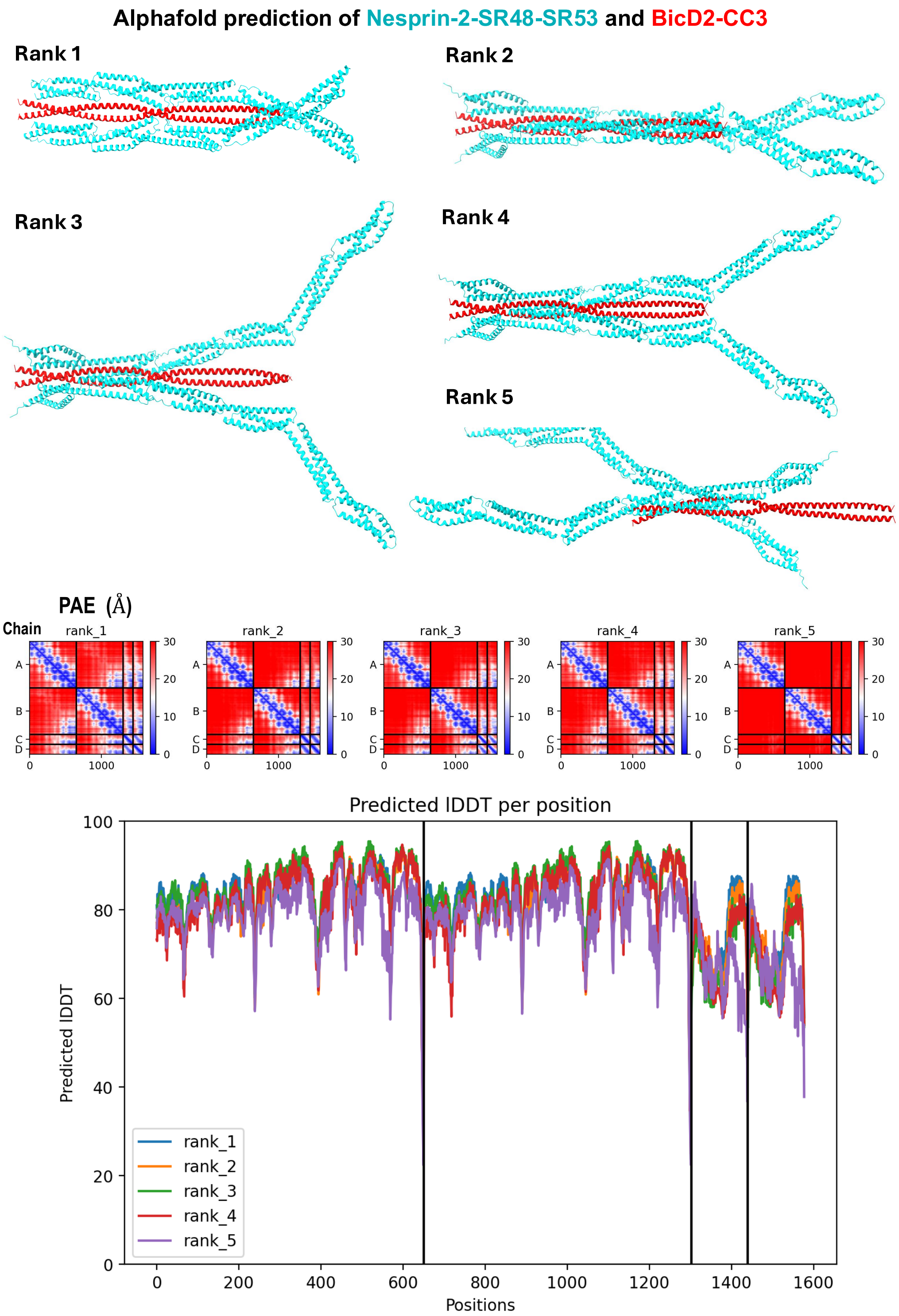

Figure S7. AlphaFold prediction of the Nesprin-2-SR48-SR53 and BicD2-CC3 complex. The five highest ranked predicted structures are shown in cartoon representation, together with the PAE and pLDDT error plots. Note that the PAE and pLDDT error suggest that the structure prediction of the additional regions in the larger complex is not reliable. See also Figure S6

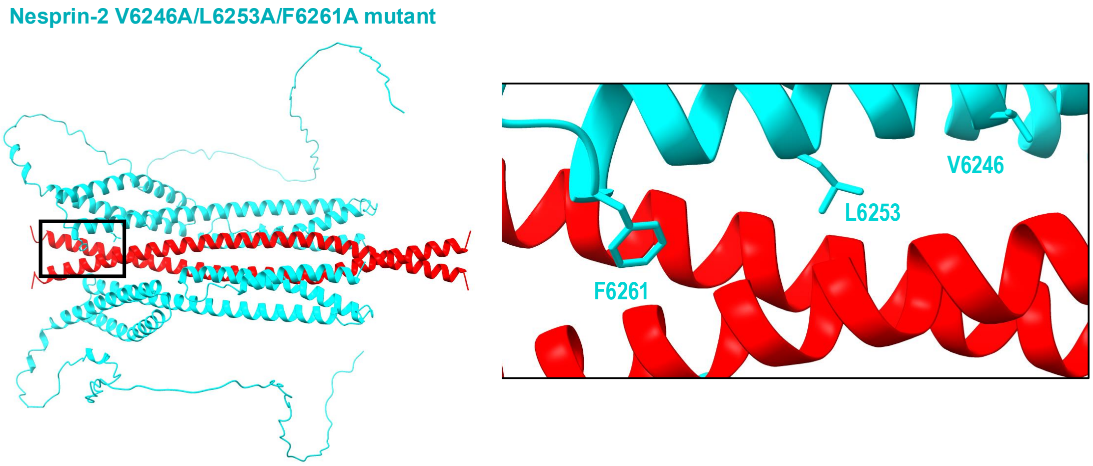

Figure S8. The Nesprin-2/V6246A/L6253A/F6261A triple mutant virtually abolishes the interaction with the BicD2-CTD. Cartoon representation of the minimal Nesprin-2/BicD2 complex. The boxed region on the left is enlarged on the right to depict the location of the mutated residues. Residues F6261, L6253 and V6246, which mediate contacts with BicD2 are shown in stick representation and labelled. See also Figure 3.

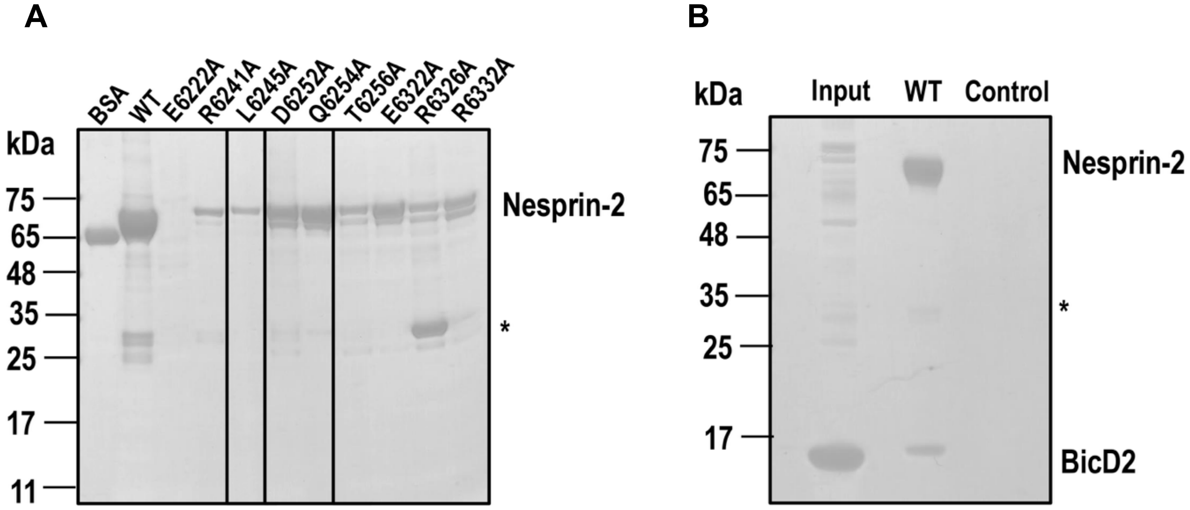

Figure S9. (A) SDS-PAGE analysis of purified Nesprin-2 mutants that were excluded from the pull-down analysis due to poor solubility. Proteins were purified from 0.5 l of cells by GST-affinity chromatography and 19 µl of the eluate was analyzed. The positions of the molecular weight standard bands are indicated. Note that the mutants have decreased yields compared to the WT, indicating low solubility. The experiment was performed in three replicates. 3 ug BSA is shown as concentration standard. The asterisk denotes GST. **(B) SDS-PAGE analysis of the negative control for the pull-down assay** (see Figures 2 and 3). The input lane shows purified BicD2 used for the pull-down. The WT Lane shows the elution fraction of a pull-down assay with the WT GST-Nesprin-2 fragment and BicD2-CTD. The control lane shows the negative control, for which BicD2 was added to the empty GST column**.** For a negative control pull-down assay with GST protein and BicD2-CTD see [21]. See also Figure 3.

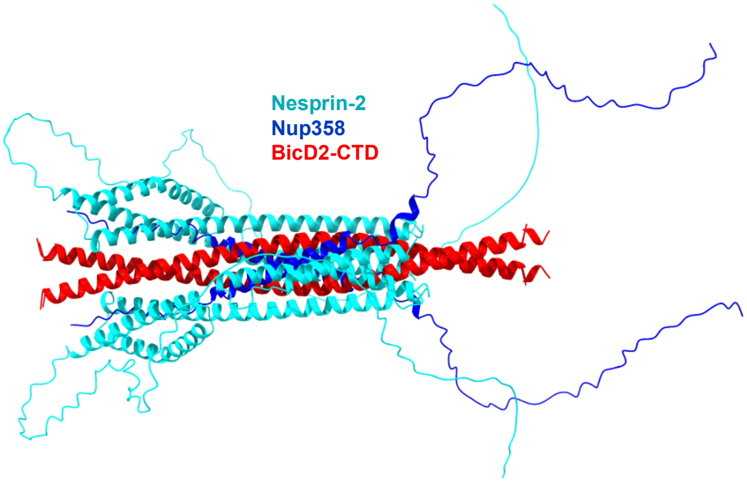

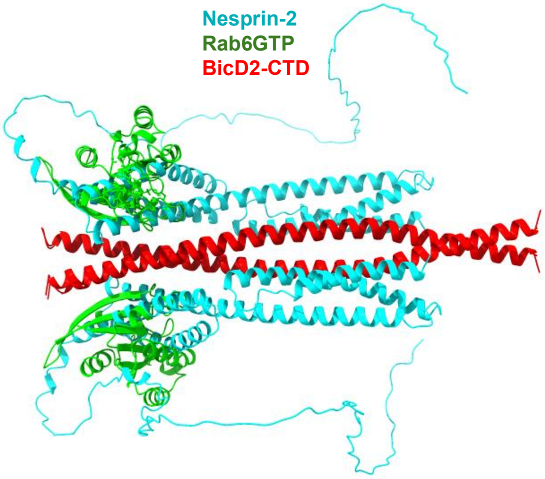

Comparison of interfaces of BicD2/cargo complexes

	Nesprin-2/BicD2	Nup358/BicD2	Rab6/BicD2
**# of contact residues**	160	209	96
**Buried surface area Å^2**	2518	3715	1535
**Secondary structure of the interacting domain (excluding BicD2)**	α-helix	α -helix<br>random coil	α-helix<br>β-sheet<br>random coil

Figure S10. The cargo adapters Nesprin-2, Nup358 and Rab6^{GTP} bind to distinct but overlapping sites on the BicD2-CTD. Least square superimposition of the structural models of Nesprin-2/BicD2 with the structural models of Nup358/BicD2 (top) and Rab6^{GTP}/BicD2 (bottom) [22–24]. See also Figure 5.

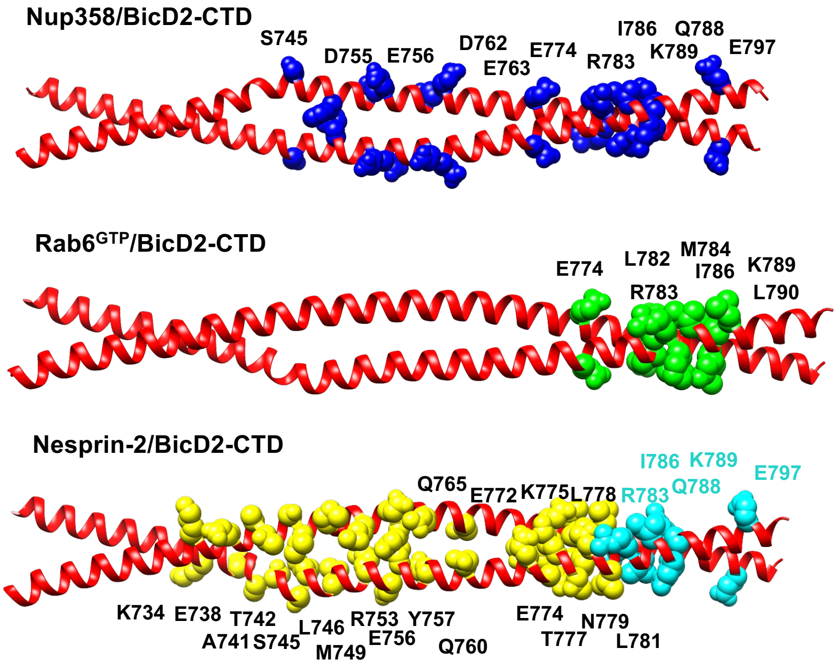

Figure S11. Comparison of binding sites for Nup358, Nesprin-2 and Rab6^{GTP} on the BicD2-CTD. The structure of the human BicD2-CTD is shown in red cartoon representation (PDB ID 6OFP)[25]. The contact residues for three cargoes that were verified by mutagenesis and binding assays are highlighted in sphere representation, labeled and colored blue for Nup358 (top panel), green for Rab6^{GTP} (middle panel) and cyan for Nesprin-2 (bottom panel) [21–24,26]. For Nesprin-2, additional BicD2 contact residues from the AlphaFold structural model are highlighted in yellow. See also Figure 5

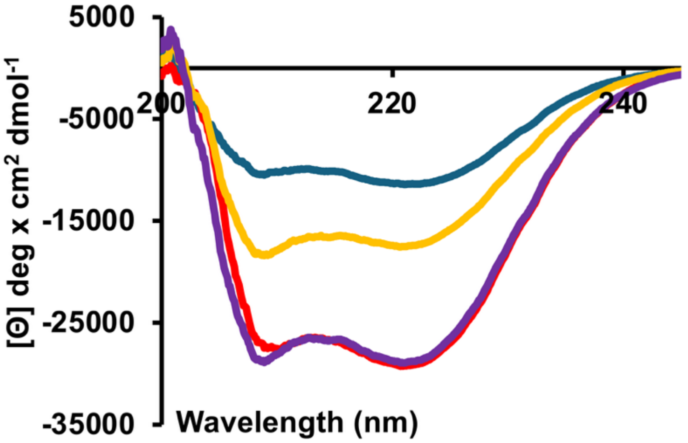

Molar ellipticity	[Θ] 208 nm (3.5 % error)	[Θ] 222 nm (5% error)
Nesprin-2/BicD2 complex	-29092 ±1018	-26224 ±1311
Nesprin-2 + BicD2	-26224 ±918	-29092 ±1455
Nesprin-2	-18147 ±635	-17402 ±870
BicD2	-11375 ±398	-10297 ±515

Secondary structure estimation from the CD spectra with BeStSel [1]

Protein	α-helix (%)	Anti-parallel β-sheet (%)	Parallel β-sheet (%)	Turn (%)	Other (%)
Nesprin-2	57.0	27.3	0	15.7	0
BicD2	21.9	24.5	1.6	16.0	36.0
Nesprin-2/BicD2 complex	49.6	28.5	15.5	6.3	0
Nesprin-2 + BicD2	49.6	28.5	15.5	6.3	0

Figure S12. The minimal Nesprin-2/BicD2-CTD complex has a similar alpha-helical content compared to the individual BicD2-CTD and Nesprin-2 fragments. A minimal Nesprin-2/BicD2-CTD complex was assembled by mixing the purified Nesprin-2 fragment lacking the intrinsically disordered AD domain (aa 6123-6352, 0.4 mg/ml) with BicD2-CTD (aa 715-804, 0.1 mg/ml). The CD wavelength scan of the assembled complex is shown in purple, overlaid with the CD wavelength scans of the individual proteins (Nesprin-2: yellow; BicD2-CTD: blue) and the sum of the CD wavelength scans of the two individual proteins Nesprin-2 and BicD2-CTD (red). For the CD experiments shown here, the Nesprin-2 fragment lacking the intrinsically disordered AD domain (aa 6123-6352 for sequence see Figure S13) was used, while for the CD experiments in Figure 4 the Nesprin-2 fragment aa 6123-6421 was used. The molar ellipticity [θ] is plotted versus the wavelength. The experiment was repeated three times, a representative experiment is shown. A table lists [θ] at 208 and 222 nm (characteristic wavelength for alpha-helical structures) including experimental error [27]. Note that [θ] at 222 nm of the complex is similar to the sum of the CD wavelength scans of the individual proteins, suggesting a similar alpha-helical content. A secondary structure estimation from the CD spectra was carried out using BeStSel [1].

Nesprin-2 aa 6123-6352 (single molecule assays, Figure S12)
MSPILGYWKIKGLVQPTRLLLEYLEEKYEEHLYERDEGDKWRNKKFELGLEFPNLPYYIDGDVK
LTQSMAIIRYIADKHNMLGGCPKERAEISMLEGAVLDIRYGVSRIAYSKDFETLKVDFLSKLPEML
KMFEDRLCHKTYLNGDHVTHPDFMLYDALDVVLYMDPMCLDAFPKLVCFKKRIEAIPQIDKYLK
SSKYIAWPLQGWQATFGGGDHPPKSDLEVLFQGPLGSRLWQKFLDDYSRFEDWLKSAERTAA
CPNSSEVLYTNAKEELKRFEAFQRQIHERLTQLELINKQYRRLARENRTDTASKLKQMVHEGNQ
RWDNLQKRVTAILRRLRYFTNQREEFEGTRESILVWLTEMDLQLTNVEHFSESDAEDKMRQLN
GFQQEITLNTNKIDQLIVFGEQLIQKSEPLDAVLIEDELEELHRYCQEVFGRVSRFHRRLTSHTPG
LDDEKEASENETMDYKDDDDKAGEAPAAAEISGHIVRSPMVGTFYRTPSPDAKAFIEVGQKVN
VGDTLCIVEAMKMMNQIEADKSGTVKAILVESGQPVEFDEPLVVIEGS

Nesprin-2 aa 6123-6421 (single molecule assays)
MSPILGYWKIKGLVQPTRLLLEYLEEKYEEHLYERDEGDKWRNKKFELGLEFPNLPYYIDGDVK
LTQSMAIIRYIADKHNMLGGCPKERAEISMLEGAVLDIRYGVSRIAYSKDFETLKVDFLSKLPEML
KMFEDRLCHKTYLNGDHVTHPDFMLYDALDVVLYMDPMCLDAFPKLVCFKKRIEAIPQIDKYLK
SSKYIAWPLQGWQATFGGGDHPPKSDLEVLFQGPLGSRLWQKFLDDYSRFEDWLKSAERTAA
CPNSSEVLYTNAKEELKRFEAFQRQIHERLTQLELINKQYRRLARENRTDTASKLKQMVHEGNQ
RWDNLQKRVTAILRRLRYFTNQREEFEGTRESILVWLTEMDLQLTNVEHFSESDAEDKMRQLN
GFQQEITLNTNKIDQLIVFGEQLIQKSEPLDAVLIEDELEELHRYCQEVFGRVSRFHRRLTSHTPG
LDDEKEASENETDIEDPREIQADSWRKRRESEEPTSPQSLCHLVPPALGHERSGCETPVSVDSI
PLEWDHTGDVGGSSSHETTRRAHSTAHCQMMKQLEDKVEELLSKNYHLENEVARLKKLVGER
MSGDKDCEMKRTTLDSPLGKLELSGCEQGLHRIIFLGKGTSAADAVEVPAPAAVLGGPEPLMQ
ATAWLNAYFHQPEAIEEFPVPALHHPVFQQESFTRQVLWKLLKVVKFGEVISYSHLAALAGNPAA
TAAVKTALSGNPVPILIPCHRVVQGDLDVGGYEGGLAVKEWLLAHEGHRLGKPGLG

Nesprin-2 aa 6123-6352 (SEC-MALS)
SPILGYWKIKGLVQPTRLLLEYLEEKYEEHLYERDEGDKWRNKKFELGLEFPNLPYYIDGDVKLT
QSMAIIRYIADKHNMLGGCPKERAEISMLEGAVLDIRYGVSRIAYSKDFETLKVDFLSKLPEMLK
MFEDRLCHKTYLNGDHVTHPDFMLYDALDVVLYMDPMCLDAFPKLVCFKKRIEAIPQIDKYLKS
SKYIAWPLQGWQATFGGGDHPPKSDLEVLFQGPLGSRLWQKFLDDYSRFEDWLKSAERTAAC
PNSSEVLYTNAKEELKRFEAFQRQIHERLTQLELINKQYRRLARENRTDTASKLKQMVHEGNQR
WDNLQKRVTAILRRLRYFTNQREEFEGTRESILVWLTEMDLQLTNVEHFSESDAEDKMRQLNG
FQQEITLNTNKIDQLIVFGEQLIQKSEPLDAVLIEDELEELHRYCQEVFGRVSRFHRRLTSHTPGL
DDEKEASENET

Figure S13. Protein sequences of the Nesprin-2 fragments used for single molecule assays, SEC-MALS and Figure S12.

SUPPORTING MOVIE LEGENDS

Movie S1. Single-molecule processivity assays of DDBN complexes with dynein, dynactin, full-length BicD2 and Nesprin-2 fragments on MTs. The movie is playing at 10× speed.

Movie S2. Single-molecule processivity assays of DDB complexes with full length auto-inhibited BicD2 on MTs. The movie is playing at 10× speed.

SUPPORTING METHODS

SEC-MALS

The Nesprin-2 fragment was purified using gel filtration as described under single-molecule experiments, and the BicD2-CTD fragment was purified as described in [28]. Prior to analysis, purified proteins were filtered (0.02 um pore size) and centrifuged in a microcentrifuge (4 °C, 21700g, 30 min). 100 ul sample was injected at room temperature onto a Superdex 200 Increase 10/300 GL column (Cytiva) that was equilibrated with 20 mM HEPES (pH 7.5), 500 mM NaCl, and 0.5 mM TCEP. The column was connected to a Wyatt Technology DAWN 8+ multiangle light scattering detector and an Optilab TrEX refractive index detector. ASTRA 6.1 (Wyatt Technology) was used to determine weight-averaged molar masses.

SUPPORTING REFERENCES

(1) Micsonai, A., Moussong, É., Wien, F., Boros, E., Vadászi, H., Murvai, N., Lee, Y.-H., Molnár, T., Réfrégiers, M., Goto, Y., Tantos, Á., and Kardos, J. (2022) BeStSel: webserver for secondary structure and fold prediction for protein CD spectroscopy. *Nucleic Acids Res 50*, W90–W98.

(2) Mertins, P., Yang, F., Liu, T., Mani, D. R., Petyuk, V. A., Gillette, M. A., Clauser, K. R., Qiao, J. W., Gritsenko, M. A., Moore, R. J., Levine, D. A., Townsend, R., Erdmann-Gilmore, P., Snider, J. E., Davies, S. R., Ruggles, K. V, Fenyo, D., Kitchens, R. T., Li, S., Olvera, N., Dao, F., Rodriguez, H., Chan, D. W., Liebler, D., White, F., Rodland, K. D., Mills, G. B., Smith, R. D., Paulovich, A. G., Ellis, M., and Carr, S. A. (2014) Ischemia in Tumors Induces Early and Sustained Phosphorylation Changes in Stress Kinase Pathways but Does Not Affect Global Protein Levels * . *Molecular & Cellular Proteomics 13*, 1690–1704.

(3) Shiromizu, T., Adachi, J., Watanabe, S., Murakami, T., Kuga, T., Muraoka, S., and Tomonaga, T. (2013) Identification of Missing Proteins in the neXtProt Database and Unregistered Phosphopeptides in the PhosphoSitePlus Database As Part of the Chromosome-Centric Human Proteome Project. *J Proteome Res 12*, 2414–2421.

(4) Sacco, F., Humphrey, S. J., Cox, J., Mischnik, M., Schulte, A., Klabunde, T., Schäfer, M., and Mann, M. (2016) Glucose-regulated and drug-perturbed phosphoproteome reveals molecular mechanisms controlling insulin secretion. *Nat Commun 7*, 13250.

(5) Mertins, P., Mani, D. R., Ruggles, K. V, Gillette, M. A., Clauser, K. R., Wang, P., Wang, X., Qiao, J. W., Cao, S., Petralia, F., Kawaler, E., Mundt, F., Krug, K., Tu, Z., Lei, J. T., Gatza, M. L., Wilkerson, M., Perou, C. M., Yellapantula, V., Huang, K., Lin, C., McLellan, M. D., Yan, P.,

Davies, S. R., Townsend, R. R., Skates, S. J., Wang, J., Zhang, B., Kinsinger, C. R., Mesri, M., Rodriguez, H., Ding, L., Paulovich, A. G., Fenyö, D., Ellis, M. J., Carr, S. A., and CPTAC, N. C. I. (2016) Proteogenomics connects somatic mutations to signalling in breast cancer. *Nature 534*, 55–62.

(6) Bouhaddou, M., Memon, D., Meyer, B., White, K. M., Rezelj, V. V, Correa Marrero, M., Polacco, B. J., Melnyk, J. E., Ulferts, S., Kaake, R. M., Batra, J., Richards, A. L., Stevenson, E., Gordon, D. E., Rojc, A., Obernier, K., Fabius, J. M., Soucheray, M., Miorin, L., Moreno, E., Koh, C., Tran, Q. D., Hardy, A., Robinot, R., Vallet, T., Nilsson-Payant, B. E., Hernandez-Armenta, C., Dunham, A., Weigang, S., Knerr, J., Modak, M., Quintero, D., Zhou, Y., Dugourd, A., Valdeolivas, A., Patil, T., Li, Q., Hüttenhain, R., Cakir, M., Muralidharan, M., Kim, M., Jang, G., Tutuncuoglu, B., Hiatt, J., Guo, J. Z., Xu, J., Bouhaddou, S., Mathy, C. J. P., Gaulton, A., Manners, E. J., Félix, E., Shi, Y., Goff, M., Lim, J. K., McBride, T., O'Neal, M. C., Cai, Y., Chang, J. C. J., Broadhurst, D. J., Klippsten, S., De wit, E., Leach, A. R., Kortemme, T., Shoichet, B., Ott, M., Saez-Rodriguez, J., tenOever, B. R., Mullins, R. D., Fischer, E. R., Kochs, G., Grosse, R., García-Sastre, A., Vignuzzi, M., Johnson, J. R., Shokat, K. M., Swaney, D. L., Beltrao, P., and Krogan, N. J. (2020) The Global Phosphorylation Landscape of SARS-CoV-2 Infection. *Cell 182*, 685-712.e19.

(7) Gnad, F., Young, A., Zhou, W., Lyle, K., Ong, C. C., Stokes, M. P., Silva, J. C., Belvin, M., Friedman, L. S., Koeppen, H., Minden, A., and Hoeflich, K. P. (2013) Systems-wide Analysis of K-Ras, Cdc42, and PAK4 Signaling by Quantitative Phosphoproteomics *. *Molecular & Cellular Proteomics 12*, 2070–2080.

(8) Sharma, K., D'Souza, R. C. J., Tyanova, S., Schaab, C., Wiśniewski, J. R., Cox, J., and Mann, M. (2014) Ultradeep Human Phosphoproteome Reveals a Distinct Regulatory Nature of Tyr and Ser/Thr-Based Signaling. *Cell Rep 8*, 1583–1594.

(9) Huttlin, E. L., Jedrychowski, M. P., Elias, J. E., Goswami, T., Rad, R., Beausoleil, S. A., Villén, J., Haas, W., Sowa, M. E., and Gygi, S. P. (2010) A Tissue-Specific Atlas of Mouse Protein Phosphorylation and Expression. *Cell 143*, 1174–1189.

(10) Mertins, P., Qiao, J. W., Patel, J., Udeshi, N. D., Clauser, K. R., Mani, D. R., Burgess, M. W., Gillette, M. A., Jaffe, J. D., and Carr, S. A. (2013) Integrated proteomic analysis of post-translational modifications by serial enrichment. *Nat Methods 10*, 634–637.

(11) Santamaria, A., Wang, B., Elowe, S., Malik, R., Zhang, F., Bauer, M., Schmidt, A., Silljé, H. H. W., Körner, R., and Nigg, E. A. (2011) The Plk1-dependent Phosphoproteome of the Early Mitotic Spindle *. *Molecular & Cellular Proteomics 10*.

(12) Olsen, J. V, Vermeulen, M., Santamaria, A., Kumar, C., Miller, M. L., Jensen, L. J., Gnad, F., Cox, J., Jensen, T. S., Nigg, E. A., Brunak, S., and Mann, M. (2010) Quantitative Phosphoproteomics Reveals Widespread Full Phosphorylation Site Occupancy During Mitosis. *Sci Signal 3*, ra3–ra3.

(13) Pan, C., Olsen, J. V, Daub, H., and Mann, M. (2009) Global Effects of Kinase Inhibitors on Signaling Networks Revealed by Quantitative Phosphoproteomics *. *Molecular & Cellular Proteomics 8*, 2796–2808.

(14) Grimsrud, P. A., Carson, J. J., Hebert, A. S., Hubler, S. L., Niemi, N. M., Bailey, D. J., Jochem, A., Stapleton, D. S., Keller, M. P., Westphall, M. S., Yandell, B. S., Attie, A. D., Coon, J. J., and Pagliarini, D. J. (2012) A Quantitative Map of the Liver Mitochondrial Phosphoproteome Reveals Posttranslational Control of Ketogenesis. *Cell Metab 16*, 672–683.

(15) Kettenbach, A. N., Schweppe, D. K., Faherty, B. K., Pechenick, D., Pletnev, A. A., and Gerber, S. A. (2011) Quantitative Phosphoproteomics Identifies Substrates and Functional Modules of Aurora and Polo-Like Kinase Activities in Mitotic Cells. *Sci Signal 4*, rs5–rs5.

(16) Hsu, P. P., Kang, S. A., Rameseder, J., Zhang, Y., Ottina, K. A., Lim, D., Peterson, T. R., Choi, Y., Gray, N. S., Yaffe, M. B., Marto, J. A., and Sabatini, D. M. (2011) The mTOR-Regulated Phosphoproteome Reveals a Mechanism of mTORC1-Mediated Inhibition of Growth Factor Signaling. *Science (1979) 332*, 1317–1322.

(17) Yu, Y., Yoon, S.-O., Poulogiannis, G., Yang, Q., Ma, X. M., Villén, J., Kubica, N., Hoffman, G. R., Cantley, L. C., Gygi, S. P., and Blenis, J. (2011) Phosphoproteomic Analysis Identifies Grb10 as an mTORC1 Substrate That Negatively Regulates Insulin Signaling. *Science (1979) 332*, 1322–1326.

(18) Klammer, M., Kaminski, M., Zedler, A., Oppermann, F., Blencke, S., Marx, S., Müller, S., Tebbe, A., Godl, K., and Schaab, C. (2012) Phosphosignature Predicts Dasatinib Response in Non-small Cell Lung Cancer *. *Molecular & Cellular Proteomics 11*, 651–668.

(19) Zhou, C., Wu, Y. K., Ishidate, F., Fujiwara, T. K., and Kengaku, M. (2024) Nesprin-2 coordinates opposing microtubule motors during nuclear migration in neurons. *Journal of Cell Biology 223*, e202405032.

(20) Zhu, R., Antoku, S., and Gundersen, G. G. (2017) Centrifugal Displacement of Nuclei Reveals Multiple LINC Complex Mechanisms for Homeostatic Nuclear Positioning. *Curr Biol 27*, 3097-3110.e5.

(21) Yi, J., Zhao, X., Noell, C. R., Helmer, P., Solmaz, S. R., and Vallee, R. B. (2023) Role of Nesprin-2 and RanBP2 in BICD2-associated brain developmental disorders. *PLoS Genet 19*, e1010642.

(22) Gibson, J. M., Cui, H., Ali, M. Y., Zhao, X., Debler, E. W., Zhao, J., Trybus, K. M., Solmaz, S. R., and Wang, C. (2022) Coil-to-α-helix transition at the Nup358-BicD2 interface activates BicD2 for dynein recruitment. *Elife 11*, e74714.

(23) Gibson, J. M., Zhao, X., Ali, M. Y., Solmaz, S. R., and Wang, C. (2023) A structural model for the core Nup358-BicD2 interface. *Biomolecules 13*, 1445.

(24) Zhao, X., Quintremil, S., Rodriguez Castro, E. D., Cui, H., Moraga, D., Wang, T., Vallee, R. B., and Solmaz, S. R. (2024) Molecular mechanism for recognition of the cargo adapter Rab6GTP by the dynein adapter BicD2. *Life Sci Alliance 7*, e202302430.

(25) Noell, C. R., Loh, J. Y., Debler, E. W., Loftus, K. M., Cui, H., Russ, B. B., Zhang, K., Goyal, P., and Solmaz, S. R. (2019) Role of Coiled-Coil Registry Shifts in the Activation of Human Bicaudal D2 for Dynein Recruitment upon Cargo Binding. *J Phys Chem Lett 10*, 4362–4367.

(26) Terawaki, S.-I., Yoshikane, A., Higuchi, Y., and Wakamatsu, K. (2015) Structural basis for cargo binding and autoinhibition of Bicaudal-D1 by a parallel coiled-coil with homotypic registry. *Biochem Biophys Res Commun 460*, 451–456.

(27) Cui, H., Ali, M. Y., Goyal, P., Zhang, K., Loh, J. Y., Trybus, K. M., and Solmaz, S. R. (2020) Coiled-coil registry shifts in the F684I mutant of Bicaudal D result in cargo-independent activation of dynein motility. *Traffic 21*, 463–478.

(28) Noell, C. R., Loftus, K. M., Cui, H., Grewer, C., Kizer, M., Debler, E. W., and Solmaz, S. R. (2018) A Quantitative Model for BicD2/Cargo Interactions. *Biochemistry 57*, 6538–6550.
